# Connectivity as a multiple: In, with and as “nature”

**DOI:** 10.1111/area.12353

**Published:** 2017-05-24

**Authors:** Timothy Hodgetts

**Affiliations:** ^1^ School of Geography and the Environment University of Oxford Oxford UK

**Keywords:** biogeography, connectivity, more‐than‐human, nature

## Abstract

Connectivity is a central concept in contemporary geographies of nature, but the concept is often understood and utilised in plural ways. This is problematic because of the separation, rather than the confusion, of these different approaches. While the various understandings of connectivity are rarely considered as working together, the connections between them have significant implications. This paper thus proposes re‐thinking connectivity as a “multiple”. It develops a taxonomy of existing connectivity concepts from the fields of biogeography and landscape ecology, conservation biology, socio‐economic systems theory, political ecology and more‐than‐human geography. It then considers how these various understandings might be re‐thought not as separate concerns, but (following Annemarie Mol) as “more than one, but less than many”. The implications of using the connectivity multiple as an analytic for understanding conservation practices are demonstrated through considering the creation of wildlife corridors in conservation practice. The multiple does not just serve to highlight the practical and theoretical linkages between ecological theories, social inequities and affectual relationships in more‐than‐human worlds. It is also suggestive of a normative approach to environmental management that does not give temporal priority to biological theories, but considers these as always already situated in these social, often unequal, always more‐than‐human ecologies.

## 1 INTRODUCTION

Connectivity is a central concept within contemporary geographic understandings of “nature”, but is understood in different (plural) ways across the discipline and in policy discourse. These differences are problematic, but not because they lead to conceptual ambiguity. After all, there is rarely confusion between the ecological connections in habitats described by biogeographers and the emotional connections between people and the natural world theorised by cultural geographers. However, I suggest that the problems arise exactly because these different types of connectivity are considered as being separate: as sharing a coincidental terminology, but pertaining to different things.

Instead of this pluralist reading, I suggest that connectivity can be productively thought as a *multiple*. The science studies theorist Annemarie Mol describes the concept of the multiple as “more than one – but less than many … [it] is not fragmented. Even if it is multiple, it also hangs together” (2002, p. 55). Multiplicity is thus differentiated from pluralism – the latter allows for many distinct realities, whereas the former addresses how such realities “*overlap and interfere with one another*” (Law, 2004, p. 61, emphasis in original). The problems of a plural, rather than multiple, understanding of connectivity are both conceptual and practical. Conceptual because the things, events and processes understood through different ideas about connectivity are themselves meaningfully connected. And practical because how these multiple forms of connectivity are enacted has significant implications for the ways in which humans engage with, protect and manage “natures”.

In this paper I trace the most influential (and distinct) articulations of connectivity within geographies of nature, and suggest how they might be re‐thought as a multiple. The remainder of this paper is divided into four parts. Section two outlines how biogeographic understandings of connectivity theorise “nature” as connected spatially and ecologically, either as structural habitat connections or in terms of landscape permeability. In part three, literatures from both conservation science and political ecology are utilised to explore the interplay of spatial, social and emotional connections with “nature”, and to show how these types of connectivity influence the need for and practice of nature conservation in different ways. In the fourth section, more‐than‐human approaches are shown to re‐imagine the ontology of connections in “natures”, drawing on new materialist sensibilities. And finally, drawing especially on these more‐than‐human literatures, I suggest that scholars and practitioners might benefit from re‐thinking these plural ideas about connectivity as a connectivity multiple. Through a brief illustrative example (the creation of wildlife corridors), I suggest that doing so foregrounds the practical and theoretical linkages between ecological theories, social inequities and affectual relationships in more‐than‐human worlds.

## 2 CONNECTIVITY IN NATURE: HOW IS “NATURE” CONNECTED?

Historically, geographic thinkers have often noted the importance of connectivity to the evolution and proliferation of wildlife (e.g. Wallace, 1869). Yet in wildlife conservation, particularly in the middle of the 20th century, the “crisis” of species extinctions led to a set of political, tactical and ecological operating logics in which the protection of bounded habitat areas took precedence over their outward connections. These protective logics were given theoretical credence through the partial application of MacArthur and Wilson's (1967) theory of island biogeography to the extinction problem (Kingsland, 2002); it was “partial” in that, of the theory's two key variables, “area” was emphasised over “isolation”. The dominance of the resulting “protected area approach” to wildlife conservation has seen a huge increase in the size and number of nature reserves globally (Chape, Harrison, Spalding, & Lysenko, 2005). Yet some influential conservation biologists argue that this is still not enough. For wildlife conservation to be effective, they suggest that half the earth's terrestrial surface needs to be designated in reserves (Wilson, 2016). In recent decades, however, developments in ecology and biogeography have also re‐emphasised the importance of ecological connections for conservation to be effective (Haila, 2002). While not disputing the need for protected areas, nor denigrating their importance, new connectivity theories address some of their shortcomings: many nature reserves are not big enough (individually) to support sustainable populations of large or wide‐ranging species (Crooks & Sanjayan, 2006), or those that persist through metapopulation dynamics (Hanski & Gilpin, 1996); and climate change means many species will need to move between reserves to survive in an uncertain future (Thomas et al., 2004).

### 2.1 Biogeography, landscape ecology, conservation biology

Informed by Forman's influential work on landscape ecology (Forman, 1995), biogeographic articulations of connectivity have been measured and defined in various ways. The main two are “structural” and “functional” forms. Structural connectivity refers to the extent to which habitats are spatially linked and contiguous, referred to as “spatial connectedness” in some accounts (Jongman & Pungetti, 2004). The most simplistic form of spatial connectivity is the habitat corridor. “Corridors are appealing”, explains one commentary,

because they purportedly maintain or restore the very connectivity that fragmentation undermines. Whether in a backyard or across a landscape, everyone can visualize a corridor and associate it with connectivity. (Anderson & Jenkins, 2006, p. 4)

Yet there is much debate in ecological literatures about their efficacy, with concerns including: whether target species actually use the corridors; the prevalence of edge effects within the corridors; the preferential facilitation of migration by generalist and invasive species; the ways in which corridors can act as a conduit for disease; and the unintended creation of sink populations, too small to survive without further immigration or habitat extension (Hilty, Lidicker, & Merenlender, 2006). The efficacy of such corridors is also a function of scale, with regional‐scale corridor schemes perhaps transcending some of the issues listed above. Alongside corridors, ideas about structural connectivity have in addition led to the development of a body of theory and policy concerned with the linkages between “networks” of habitats (Jongman & Pungetti, 2004). This line of analysis has been applied to the design of protected area networks to maximise structural connectivity (Saura, Estreguil, Mouton, & Rodriguez‐Freire, 2011).

By contrast, functional connectivity (sometimes termed “ecological” connectivity) focuses not on habitat linkages but on species’ movements. Some authors instead advocate the terminology of “landscape permeability” for functional/ecological notions. Soule et al. suggest the latter is a more helpful term because:

(1) it suggests the importance of dynamic processes; (2) it reminds us of the species‐specific nature of obstacles to movements, and (3) it requires conservationists to consider the landscape (including the “matrix” of unprotected country) as a whole, rather than focusing on narrow, defined corridors. (2006, p. 651)

Functional connectivity is also sometimes defined more broadly, encompassing not only organismal movement but also referring to the extent to which a landscape or habitat is adequately connected so as to facilitate ecological processes to occur (Crooks & Sanjayan, 2006). Structural and functional understandings about connectivity have increasingly influenced conservation policy and practice over the past few decades: from small‐scale habitat linkages, to agricultural schemes designed to facilitate landscape permeability (Lawton et al., 2010), to regional scale habitat connections that link large protected area networks such as the Yellowstone to Yukon “Y2Y” conservation scheme (Worboys, Francis, & Lockwood, 2010).

## 3 CONNECTIVITY WITH NATURE: HOW TO RE‐CONNECT PEOPLE AND NATURE?

While biogeography has focused on the question of “how nature is connected”, the wider conservation movement has also become increasingly concerned with the idea that *people* have become *disconnected* from nature. In the public pronouncements and writings of influential conservationists (e.g. Wilson, 2016), government reports (e.g. HM Government, 2011), and popular non‐fiction texts (Louv, 2005), a discourse around people's disconnection from nature can be clearly traced. Specifically, this line of thought links the destruction and degradation of “nature” with the increasing alienation and detachment of people from nature (Pyle, 2003). If only people can be re‐connected, goes the narrative, then support for wildlife conservation and environmental protection will increase significantly.

In some versions, simply getting people to visit or live near to (spatially disconnected) “nature” will ignite an emotional connection. For instance, Wilson's concept of “biophilia” suggests that humans have an innate love for life that can be sparked through engaging with the natural world (Wilson, 1984). Less reliant on universal assumptions about love, however, there is a significant body of work that looks at how emotional connections with species and ecologies can facilitate conservation action, particularly through the affective agency of charismatic “flagship species” (Lorimer, 2007). And taking somewhat similar logics into the policy realm, the UK Government's white paper *The natural choice* sets out policy measures that build on emotional logics to create a social form of connectivity with “nature”. The policy document pronounces:

Our ambition is to strengthen the connections between people and nature. We want more people to enjoy the benefits of nature by giving them freedom to connect with it. Everyone should have fair access to a good‐quality natural environment. (HM Government, 2011, p. 44)

The subsequent chapter (a quarter of the document) sets out how this can be achieved. But note also the utilitarian logics at work in this example: it draws on the assumption that nature provides “benefits” to humans, which are used to justify the policies that seek to “reconnect” people.

### 3.1 Socio‐ecological systems

The logic of re‐connection can thus be articulated in different ways by approaches that analyse people–nature connections at an aggregated, society‐wide scale. Therein, the focus shifts from personal emotional connectivity (that underpins attitudes towards “nature”) and towards the myriad ecological relationships that underpin modern societies. In particular, researchers at the Stockholm Resilience Centre and elsewhere have developed social‐ecological systems (SES) approaches to mapping, monitoring and managing the myriad links between societies and the ecologies on which they depend (Berkes, Colding, & Folke, 2008). The aim of such work is to make visible the connections between nature and society, to emphasise the specificities of how the latter relies on the former, and to thus guide environmental policy for the benefit of humankind. However, such approaches are not always practically successful, even assessed on their own terms, when it comes to promoting connectivity. For example, recent work has pointed to the risks generated when structural and ecological connections are framed as “green infrastructure” with multiple societal and ecological benefits: the ecological benefits may fail to materialise and/or they may be subsumed by other societal (economic) needs (Garmendia, Apostolopoulou, Adams, & Bormpoudakis, 2016).

### 3.2 Political ecology

Some of the modes of social/political reconnection suggested in contemporary policy, especially those that focus predominantly on the economic values of nature to human populations, have been further questioned by political ecologists (Büscher, Sullivan, Neves, Igoe, & Brockington, 2012). These literatures offer a powerful critique of the re‐connection discourse as it has been captured by capitalist logics and turned towards a neoliberal agenda (Castree, 2008). In practice, valuing nature solely through its economic usefulness to humans disconnects all manner of ecological processes, obscures plural human values and threatens more‐than‐human affective connections (Büscher et al., 2012). Indeed, political ecologists have often drawn attention to the ways in which various concepts created by conservation biologists have been mobilised to support questionable forms of governance regime in the name of environmental management (Pincetl, Jonas, & Sullivan, 2011). Such work has also drawn attention to the social inequalities created, perpetuated and/or amplified through the spatial practices of conservation aimed at facilitating ecological connectivity (Brockington, Duffy, & Igoe, 2008; Evans, 2007). In this mode of thinking, rather than reifying connectivity as an “ecological positive”, connectivity conservation practices are assessed for the (often negatively‐valued) ways in which they produce neoliberal natures.

## 4 CONNECTIVITY AS SOCIONATURES: RE‐THINKING CONNECTIVITY IN NON‐BINARY FORMS

The notion of “re‐connecting people with nature” can, of course, be problematised on multiple grounds. Both the idea of a singular “nature” and the notion of a nature that exists in binary relationship to culture have been much critiqued (Castree, 2005). A body of work in recent human geography thus traces how conservationists and wider environmental publics engage with more‐than‐human life (to use a differently freighted term), in a way that enacts and affirms emotional *and* social connections without prioritising anthropocentrism.

### 4.1 More‐than‐human geographies

More‐than‐human approaches draw attention to the diverse ways in which lay publics and conservationists alike “learn to be affected” by non‐human organisms through actively seeking practical, affective and emotional connections with non‐human life (Hinchliffe, Kearnes, Degen, & Whatmore, 2005). These authors (and others working in a similar vein) have drawn on a variety of philosophical resources in order to theorise the affective and emotional connections that operate through interspecies communication (Adams, 2016), empathy (Despret, 2013), careful forms of anthropomorphism (Johnston, 2008) or through modes of multi‐species companionship that pay attention to difference (Haraway, 2008). Although often biased towards familiar mammal species, similar lines of thought have also extended attunement further: for example, towards plants (Brice, 2014) and even parasites (Lorimer, 2016). Geographers have also, albeit less frequently, emphasised the forms of detachment that characterise some more‐than‐human relations (Ginn, 2014). Emerging at a similar time, and with much cross‐fertilisation, non‐representational approaches in cultural geography (Thrift, 2008) have contributed to this framework for thinking about attunement to more‐than‐human natures, emphasising in particular the pre‐cognitive, affective forms of connection and disconnection that register in (human) bodies as emotion (McCormack, 2013). And the wide influence of actor‐network theory and other variants of relational thought in cultural geography has facilitated theorising how these various affective and emotional connections coalesce in particular situations to create what Latour (2005) terms “the social” – that is, a heterogeneous collection of actors, contingently connected through affective means. Indeed, the development of more‐than‐human geographies in recent years is both part of and informed by this broader relational turn.

The starting points of the more‐than‐human project are similar in some ways to those of work in the socio‐ecological systems paradigm. However, the paths subsequently taken are markedly different. The theoretical impulse behind much relational work is not descriptive but radical: showing how particular situations coalesce through specific connections facilitates re‐imagining them, and perhaps re‐configuring them, through alternative connections (for example, Latour, 2005). Thus, in the influential work *When species meet*, Haraway suggests that attending to forms of attunement between (some) humans and (some) animals can facilitate a conversation about how such connections might be nurtured, built on, expanded (Haraway, 2008). Much recent work in more‐than‐human geography takes up this challenge to build an ethics on such multi‐species connections (Lorimer, 2012). Importantly, such an ethics would be less anthropocentric than that elucidated in SES‐style work. Connectivity, in this register, is thus less about the attachment of separates, and more about living with multiplicity.

### 4.2 More‐than‐human multiplicity

The wide collection of more‐than‐human work only touched on above has thus tended to focus on the connections between “humans” and “nature” not as a lack that requires fixing nor as a system that requires human management (as in the discourses/conceptualisations noted in previous sections), but instead as an empirical event that requires recognition and ethical reflection. The emphasis is thus not on diagnosing a disconnection “between people and nature”, but on illuminating the types, forms and intensities of diverse and heterogeneous connections that exist within and between humans and all manner of non‐humans. This style of thinking is thus most aligned with (and indeed often draws directly on) the notion of multiplicity utilised in this paper.

## 5 CONNECTIVITY MULTIPLE

The aim of the taxonomy of geographical connectivity (Table 1) is not simply to map existing plural definitions, but serves as a precursor to demonstrating the connections between these understandings of connectivity re‐considered as a multiple. That is, identifying the usual ways in which “plural” ideas about connectivity are involved or invoked in particular situations affords analysis of where and how they are, in practice, enacted together. This final section briefly traces an example, selected for its illustrative power, of the multiplicity of connectivity practices: the creation of habitat corridor connectivity schemes.

**Table 1:** A summary taxonomy of the “plural types” of connectivity

Types of connectivity	Field(s)	What is connected?
Spatial/structural *and*	Biogeography, landscape ecology, conservation biology	Habitats *or*
Ecological/functional		Species (mobilities)
Emotional/affective	Conservation biology or cultural geography	“People and nature” or “people as nature”
Social (economic)	Socio‐ecological systems	Ecological, political and economic processes, understood through systems logics
Social (equity)	Political ecology	Ecologies, power and equity
Social (more‐than‐human)	More‐than‐human geography	All manner of actors with agency, not limited to humans

### 5.1 Habitat corridor connectivity schemes

The recognition of the need for connectivity within and between habitats, informed through biogeographic and landscape ecological understandings, has led to a profusion of conservation schemes targeting the creation or protection of habitat corridors in recent decades (Worboys et al., 2010). These corridors can take diverse forms: local‐scale habitat creation schemes to link fragmented areas, urban greenways that facilitate mobility for non‐human animals through anthropogenic landscapes (and humans too), or linear extensions of protected areas to link reserves into spatially connected networks (Jongman & Pungetti, 2004).

Earlier, I noted some of the ecological uncertainties inherent in habitat corridor connectivity schemes. However, all such schemes are enacted in social worlds, and their social impacts can be problematic too. Political ecologists have been quick to emphasise the inequities that may result. For example, the creation of urban wildlife corridors might be utilised within capitalist planning regimes to facilitate particular “natures”, developed and spatially arranged for the benefit of powerful groups in society (Evans, 2007). Furthermore, when corridors are imposed and enforced against local people's wishes, they can be critiqued on similar grounds to protected areas that operate through excluding local communities; this is a particular concern in the Global South, where accusations of colonialism in conservation continue to concern (Brockington et al., 2008). Considered from an economic systems perspective, corridor schemes can involve expensive land purchases, restrictive land use regulations or have an “opportunity cost” in terms of foregone development to economically minded publics or policy‐makers. On a different tack again, curating support for corridor connectivity schemes depends on the kinds of social and emotional connections traced out in previous sections, and generating such support is neither automatic nor guaranteed. Corridor schemes thus tend to rely in practice on carefully aligning ecological, political and socio‐economic interests towards similar ends: involving local communities, and connecting economic and political support at multiple scales and across ecological systems marked by fragmented land tenure and national borders (Jongman & Pungetti, 2004). Of course, contemporary conservationists are well aware of the need to consider “societal factors” and generate support when designing and implementing conservation schemes.

However, the important point here is not simply a call to take account of “social factors” when producing corridors, but to consider connectivity as a multiple from the outset. While each of the perspectives on connectivity traced above provides an important analytical “cut”, enabling consideration of particular aspects of corridor schemes as “connectivity conservation”, in practice none of these forms of connectivity are enacted in separate realms. They work together, “always already”, as a multiple. Too much analytical reductionism does violence to this multiplicity, and in conservation practice facilitates (through obscuring these connections) the sorts of inequities highlighted by political ecology. To be clear: re‐thinking connectivity as a multiple therefore also has a practical implication (as well as analytical power) in the case of corridor schemes. It suggests moving from a situation where conservationists decide a habitat corridor is necessary for a particular ecological goal (and then sets about aligning the social, economic and political support necessary to achieve the corridor's creation), to a different approach whereby none of the “types” of connectivity is given temporal priority. Instead, the connectivity multiple including ecological linkages, social relations and affectual connections could (or perhaps even should) be considered holistically and concurrently in environmental management.

Re‐thinking connectivity as a multiple is not without challenges. For example, recognising the need to link multiple modes of connectivity in conservation planning tools that have hitherto focused simply on biogeographic conceptions, recent work has attempted to link ecological models of habitat connectivity with socio‐ecological models of implementation capacity and likely community support (Guerrero & Wilson, 2016). Such models can be and are linked to the forms of social analysis captured in the socio‐ecological systems approach, and could conceivably be linked also to spatially referenced data on people's attitudes, practices and even ethical sensibilities towards more‐than‐human life. While this would seem to be consonant with re‐thinking connectivity as a multiple, however, the risk is that it results in a crude and problematic form of social science, marked by misplaced attempts to shoe‐horn incommensurable concerns into cost‐benefit analyses. Returning to Mol's definition of the multiple is useful here: it is less than many (i.e. not plural), but also more than one (i.e. not reducible to a singularity). Forms of modelling that rely on producing singularities are unlikely to do justice to that multiplicity.

## 6 CONCLUSIONS

Connectivity has been understood in a plurality of ways, both in geographic theory and in conservation practice. In this paper I suggest connectivity can, and perhaps should, be better conceptualised as a multiple. The plural theories about spatial, functional, emotional and social connections are not simply reflective academic explanations; they have often had a significant influence on how conservation activities proceed. Yet they are often considered in isolation. This paper suggests that attending to connectivity as a multiple can serve as a useful analytical tool (for tracing the links in conservation practices between ecological theories, social justice concerns and more‐than‐human affectual connections with the material world) as well as a normative guide towards conservation practice. After all, how people understand ecological connections in nature, emotional connections with nature and social connections as nature combines to suggest specific, and sometimes starkly different, approaches towards environmental management.

A final example serves to demonstrate the wider importance of this analytical approach, and the stakes involved when forms of connectivity are promoted and enacted. At present, there is a passionate and vociferous debate occurring within the wildlife conservation movement about the most appropriate form of emotional and social connectivity to promote. On one side is a collection of self‐styled “traditional” conservationists, for whom the protection of wildlife is a moral imperative that derives from the intrinsic value of non‐human life, and for whom emotional connections with nature are the route to generating this ethical sensibility more widely in society (Wuerthner, Crist, & Butler, 2015). For the traditionalists, at least as caricatured in recent debates, the key connectivity assumptions are: (i) spatial/structural connectivity is paramount, and needs to be enacted in corridors between large protected areas from which human intrusion is minimised or fully restricted (and from which invasive species are to be forcibly excluded); (ii) emotional connectivity is the wellspring of ethical support, but best gained at a distance; and (iii) social connectivity of a more‐than‐human flavour rests on eco‐centric values. On the other side are advocates of the “new conservation” a movement that focuses on the economic benefits of nature to human beings, and that advocates policy instruments including the “ecosystems services” approach (Kareiva, Lalasz, & Marvier, 2012). For the new conservationists, again as caricatured, the connectivity assumptions are: (i) functional connectivity is emphasised over structural connectivity, and is best achieved by facilitating landscape permeability in lands of differing human influence (and their “novel”, hybrid ecologies); (ii) emotional connectivity can be generated through an appeal to people's self‐interests (including their wallets) as well as their hearts; and (iii) social connectivity is a function of economic value rather than deriving from more‐than‐human sensibilities.

The first approach promotes a vision of “human‐influenced” areas being strictly demarcated from “natural” areas, and relies on spatial and functional forms of connectivity to connect protected areas, while simultaneously disconnecting humans from purified places. Humans are thus “reconnected” with nature by protecting it from them. The second facilitates functional connectivity throughout diverse landscapes and ecosystems (some more human‐influenced, others less so). Humans are connected “as” nature, and adopt the conscious role of player‐manager in environmental management. Of course, both of these positions are caricatured to an extent, and recent research suggests widespread support among a majority of conservationists for a third position: somewhere simultaneously between and beyond the two extremes, not enamoured with capitalist conservation, but open to working through facilitating permeability in human‐managed landscapes (Holmes, Sandbrook, & Fisher, 2016). Nevertheless, the different ways in which this debate imagines the multiplicity of connectivity is informative. Adopting any of these approaches will lead to conservation being enacted differently, with significant consequences for all involved.
